# Simultaneous feeding of calcium butyrate and tannin extract decreased the incidence of diarrhea and proinflammatory markers in weaned piglets

**DOI:** 10.5713/ab.21.0011

**Published:** 2021-06-23

**Authors:** Camila Demarco Maito, Antonio Diego Brandão Melo, Angela Cristina da Fonseca de Oliveira, Jansller Luiz Genova, Jair Rodini Engracia Filho, Renata Ernlund Freitas de Macedo, Kelly Mazutti Monteiro, Saulo Henrique Weber, Astrid Koppenol, Leandro Batista Costa

**Affiliations:** 1 Graduate Program of Animal Science, School of Life Sciences, Pontifícia Universidade Católica do Paraná, Curitiba, Paraná 80215-901, Brazil; 2 Impextraco NV, Heist-op-den-Berg, Antwerp 2220, Belgium

**Keywords:** Antimicrobial, Immune-expression, Intestinal Histology, Organic Acid, Phytogenic, Weaning Piglets

## Abstract

**Objective:**

This study was conducted to investigate the effect of associating calcium butyrate with tannin extract, compared to an antimicrobial on the growth performance, incidence of diarrhea, intestinal histology, immune-expression of cyclooxygenase-2 (COX-2) and tumor necrosis factor α (TNF-α) in piglets.

**Methods:**

Seventy-two piglets (36 barrows and 36 gilts) weaned at 28±2 d and initial body weight of 7.17±1.07 kg were allocated to 3 treatments in a randomized complete block design with 8 replicates per treatment and 3 animals per experimental unit. Treatments were composed of NC, negative control: basal diet without additives; PC, positive control: basal diet + 40 mg/kg of colistin sulfate; or BT, basal diet + calcium butyrate + tannin extract. The butyrate and tannin inclusion levels were 0.15% in the pre-starter phase and 0.075% in the starter phase. Incidence of diarrhea was monitored daily, and on d 14 and 35 of experiment, 1 animal from each experimental unit was slaughtered to collect intestinal samples.

**Results:**

No significant differences were observed for growth performance. The butyrate- and tannin-based additive resulted in reduced (p<0.05) incidence of diarrhea in piglets during d 1 to 14 and d 1 to 35 in comparison with the other treatments. Piglets that consumed the diet containing the calcium-butyrate and tannin showed a lower (p<0.05) crypt depth in the duodenum than those receiving the NC treatment at 14 d of experimentation. The BT treatment provided a lower (p<0.05) immune-expression of COX-2 at 14 d and TNF-α at 35 d in the duodenum.

**Conclusion:**

Association between calcium butyrate and tannin extract resulted in a significant decrease in the incidence of diarrhea and inflammatory process in the duodenum of piglets. Therefore, calcium-butyrate combined with tannin could be a part of an alternative program to reduce the use of antimicrobials in the diet of weaned piglets.

## INTRODUCTION

During the weaning period, pigs undergo several stressors such as feeding changes, separation from the mother and litter, change of environment and exposure to new pathogens. Those factors, coupled with insufficient enzyme activity and reduction of absorptive capacity, with consequent alterations in intestinal histology, directly affect the performance of these animals.

In addition to its function in the absorption and metabolization of nutrients, the intestine also plays an important role in the immune system, as it contains about 70% of the body’s immune cells [1]. The intestine acts as a physical barrier that prevents the passage of pathogens, antigens and bacterial toxins into the systemic circulation.

Therefore, the use of antimicrobials as performance enhancers added to the diet of these animals is aimed at ensuring their intestinal health, adequate maintenance of the immune system, and consequently the guarantee of good production performance. Regulation (EC) no. 1831/2003 of the European Parliament of September 22/2003 banned the use of antimicrobials as food additives throughout the EU on January 1st, 2006. Brazil, like the EU, has been following this trend and restricting the use of performance-enhancing antibiotics.

Thus, alternative additives to performance-enhancing antibiotics, such as organic acids and “plant extracts” should be investigated. Butyrate is a salt of a short-chain fatty acid produced in the colon by the gastrointestinal microbiota [2]. This fatty acid has antimicrobial properties, stimulates cell epithelial proliferation, acts as an anti-inflammatory agent and assists in intestinal motility [2,3].

In recent years, tannin extract, formerly known only for its antinutritional factor in monogastric animals, has presented antimicrobial, anthelmintic, anti-inflammatory properties and antioxidant capacity [4]. However, there are no studies on the association of calcium butyrate with tannin extracts in piglet feeding.

Therefore, the objective of this study was to evaluate the combined potential of these compounds in the replacement of performance-enhancing antimicrobials on growth performance, incidence of diarrhea, intestinal histology, and immune-expression of the cyclooxygenase-2 (COX-2) and tumor necrosis factor α (TNF-α) of piglets in the nursery phase.

## MATERIALS AND METHODS

The experiment was carried out at the swine research unit located at Farm Rio Grande, Paraná, Brazil (25°39′27″ S, 49BB°18′29″ W, 910 m above sea level).

### Animal care

The present study was approved by the Ethic Committee on the Use of Animals (Comitê de Ética no Uso de Animais, CEUA) of the Pontifical Catholic University of Paraná (Pontifícia Universidade Católica do Paraná, PUCPR), Brazil (approval no. 875A).

### Animals, housing, experimental diets, and management

Seventy-two piglets (36 barrows and 36 gilts) in the nursery phase at an initial age of 28±2 d and an initial body weight of 7.17±1.07 kg, were allocated to 3 treatments in a randomized complete block design with 8 replicates per treatment and 3 animals per experimental unit. The animals were weighed at the beginning of the experiment and their weight and sex were used to distribute them equally into the different blocks.

The following experimental treatments were tested: negative control (NC): basal diet without the addition of performance-enhancing additives; positive control (PC): basal diet + 40 mg/kg of colistin sulfate; and BT: basal diet + calcium-butyrate and tannin based additive (Impextraco NV, Heist-op-den-Berg, Belgium) (inclusion levels: pre-starter phase, 0.15%; starter phase, 0.075%). The butyrate- and tannin-based additive combines coated calcium butyrate (90%) with tannin extract from *Castanea sativa* (10%), known as sweet chestnut. At an inclusion of 1.5 kg/ton this results in 1.35 kg coated calcium butyrate per ton of feed and 0.15 kg tannin extract per ton of feed in the pre-starter phase. At an inclusion of 0.75 kg/ton this results in 0.675 kg coated calcium butyrate per ton of feed and 0.075 kg tannin extract per ton of feed in the starter phase. The additives were microencapsulated to be added to the piglet diet. Calcium-butyrate was microencapsulated with a coating that contains vegetable fat and the natural extract was added to the coated butyrate product.

Animals were housed in suspended nursery pens (1.2 m ×1.6 m) with a plastic floor, manual feeders and nipple drinkers. The piglets received the pre-starter diet (from the 1st until the 14th d), the starter diet (from the 14th until the 35th d) and water *ad libitum*. The experimental period was 35 d.

The experimental diets (Table 1) were isonutritive, differing only with regard to the inclusion of the evaluated products, and were composed mainly of corn, soybean meal and vitamins-minerals, which were added to meet the nutritional requirements of the species according to the recommendations of Rostagno et al [5].

### Growth performance, incidence of diarrhea, and intestinal health

All feed supplied, orts and wasted portions were weighed to calculate average daily feed intake (ADFI). The animals were weighed at 14 and 35 d of the experiment to calculate average daily gain (ADG), and gain to feed ratio (G:F).

Incidence of diarrhea (ID) was monitored daily by assigning scores of 0 for normal feces; 1, pasty feces; 2, soft feces (creamy); and 3, aqueous feces; according to the procedure described by Salles et al [6]. Scores of 2 and 3 were considered diarrhea and the ID (%) was calculated in relation to the total number of feces counted in the pen.

At 14 d of the experiment, one animal from each experimental unit (3 piglets/pen) was slaughtered, totaling 8 piglets/treatment. On d 35 of experiment, one animal from each experimental unit (2 piglets/pen) was slaughtered, that is, one of the 2 piglets in the pen, totaling 8 animals/treatment. The chosen animal was that whose body weight was closest to the body weight average in each block on d 14 and 35 of experiment. The animals were slaughtered (16 animals/treatment) after a 12-h (water-only) fasting period to collect samples for histological and morphological analyses of the intestinal epithelium.

The animals were slaughtered in a commercial slaughterhouse subjected to the State Sanitary Inspection, according to the standards of humane slaughter recommended for animal welfare, consisting of desensitization by electrocution followed by bleeding (by sectioning the large vessels that emerge from the heart).

### Intestinal morphology

#### Structural analysis - optical microscopy

Immediately after slaughter, fragments approximately 5 cm long were collected from the duodenum (collected at 15 cm from the pyloric sphincter) and from the jejunum at the distal part (collected approximately 150 cm from the ileocecal junction). The samples were stored in a 10% formaldehyde solution and subsequently processed and stained with hematoxylin and eosin, according to the methodology of Touchette et al [7].

The stained slides were viewed and photographed using an AxioCam MRC camera (Carl Zeiss, Göttingen, Germany) coupled to the Axio Scope A1 microscope (Carl Zeiss, Jena, Germany). The Axionvision Se 64 software (Carl Zeiss, Thornwood, NY, USA) was used to measure the height of 12 villi, respective crypt depths and villus width and to calculate the villus height:crypt depth ratio.

#### Ultrastructural analysis - scanning electron microscopy

In the same region where fragments were collected for structural analysis, another segment (5 cm) of the duodenum and jejunum was removed and fixed in Karnovsky solution (2.5% glutaraldehyde and 0.1 M sodium trihydrate cacodylate) for one hour. It was then sectioned into 0.25-cm^2^ pieces (0.50× 0.50 cm) and maintained in the same solution until the drying process, which was carried out with hexamethyldisilazane for 10 min. Subsequently, the samples were subjected to scanning electron microscopy analysis [8]. In the images obtained by scanning electron microscopy, villus density was measured in an area of 1,110,619.52 μm^2^ using the scanning electron microscope VEGA3 LMU (Tescan, Brno, Czech Republic). To count the villi, 3 photos were selected per replicate.

### Immunohistochemistry

The same samples used for structural analysis were used to create the blocks via the TMA technique (tissue microarray) [9]. Paraffin blocks containing 24 sample fragments were made to subsequently prepare the slides.

COX-2 immune expression was assessed using the polyclonal anti-Cox-2 antibody (Dako, Glostrup, Denmark). Afterwards, the slides were scanned on the Axio Scan.Z1 scanner (Carl Zeiss, Jena, Germany) and analyzed using Image Pro Plus 4 software (Media Cybernetics Inc., Rockville, MD, USA). The percentage area immunolabeled with COX-2, in μm^2^, was calculated by evaluating 7 images for each replicate according to the methodology of Costa Filho et al [10].

To identify TNF-α in the fragments, the anti-TNF-α primary antibody (ABCam, Cambridge, UK) was used in the preparation of the slides. The TNF-α-positive cells were counted using images obtained from the Olympus BX40 microscope with a 40× objective lens. Five random fields were photographed for both segments (duodenum and jejunum) in each replicate, and subsequently the mean count of immunolabeled cells was obtained.

### Statistical analysis

Growth performance, intestinal morphology and immunohistochemical analysis data were subjected to the Shapiro-Wilk test for normality evaluation and the Levene test to evaluate homogeneity of variances. The statistical model included the fixed effect of treatment (experimental diets) and block as a random effect. When those requirements were met, the effect of treatments on the evaluated variables was checked by analysis of variance. For the immunohistochemistry, Tukey’s post-hoc test was performed to compare the treatments. To determine possible differences between the treatments on the ID, the scores obtained during the experimental period were subjected to the Chi-square test. Statgraphics Centurion XVI statistical software was used and the significance level of 5% (p<0.05) was adopted for all analyzed variables.

## RESULTS

### Growth performance and incidence of diarrhea

There was mortality of one piglet from the NC treatment during the entire experimental period. Body weight at 14 and 35 d, ADG, ADFI, and G:F ratio were not influenced by the treatments (Table 2). Butyrate- and tannin-based additive resulted in reduced (p<0.05) ID in piglets during the periods from 1 to 14 d and from 1 to 35 d in comparison with the other treatments.

### Intestinal morphology

There was no difference between the treatments regarding villus height, villus height:crypt depth, villus width and villus density in the duodenum and jejunum of piglets at 14 and 35 d of the experiment (Table 3). However, the animals from the BT treatment showed a lower (p<0.05) crypt depth in the duodenum than those receiving the NC treatment at 14 d of experimentation.

### Immunohistochemistry

In the duodenum, the animals receiving the BT treatment exhibited a lower (p<0.05) percentage of cells stained for COX-2 in relation to the animals fed the NC diet at 14 d of experimentation (Table 4). In addition, the animals in the PC treatment group exhibited a lower percentage of cells stained for COX-2 than the animals in the BT and NC groups (p<0.05) at 35 d in the jejunum. For the analysis of TNF-α, a difference was observed (p<0.05) in the duodenum at 35 d of experimentation, in which the animals receiving the BT treatment showed lower cellular expression when compared to the piglets receiving the NC treatment.

## DISCUSSION

### Growth performance

High tannin concentrations worsen the appeal of the feed due to its astringent taste, an event leading to lower feed intakes. However, in the performance analyses, there were no significant differences among the treatments. The present findings corroborate those described by Biagi et al [11], who provided different concentrations of tannin (0.113%, 0.225%, and 0.450%) to weaned piglets and observed no significant effects on BW at 56 d of age, ADG or ADFI. Boas et al [12] also found no significant difference in BW at 66 d of age, ADFI, ADG, and G:F in animals that received a blend of organic acids (0.5%), sodium butyrate (0.1%) and the association of organic acids and sodium butyrate, compared to the control treatment.

Zeng et al [13] used a blend of essential oil containing thymol and cinnamaldehyde (0.025%) in piglet diets and observed better results of ADG and G:F when compared to the NC treatment. The authors associated these results with several factors, such as improvement of the apparent digestibility of the diet, improvement of intestinal morphology in the jejunum and composition of the intestinal microbiota.

The effect of antimicrobials is proportional to the challenges to which animals are subjected. In this study, the animals were housed in previously disinfected experimental facilities, which were closed and kept empty for 50 d before receiving the animals. These sanitary conditions may have reduced environmental contamination and may indicate that the animals were under a low immunological challenge. It is known that the environmental conditions of commercial farms differ from those of experimental farms, in which the challenges imposed on pigs are intensified by different management strategies, microbial concentrations and population densities.

These may be possible explanations for the absence of significant results regarding the performance of animals that received the different additives. Costa et al [14] also commented that supplying highly digestible diets may decrease the antimicrobial potential of the additives, because these diets result in little substrate available for bacterial growth, thus limiting the development of microorganisms in the gastrointestinal tract. The piglets of the present study received complex and highly digestible diets, which may also be an explanation for the lack of positive results regarding animal performance.

### Incidence of diarrhea

The animals fed the BT treatment showed a significant reduction in ID compared to NC and PC groups. The present data corroborate those of Zeng et al [13], who reported reduced ID in piglets fed diets with a blend of essential oils containing 0.025% cinnamaldehyde and thymol. Cairo et al [15] studied 0.05%, 0.10%, or 0.15% Brazilian red pepper essential oil in the diet of weaned pigs and observed that pigs that received the diet 0.15% of the oil showed the lowest ID. However, Silva Júnior et al [16] evaluated a blend of different plant extracts and benzoic acid (0.3%), with or without addition of colistin sulfate (40 ppm) for weaned piglets, and did not observe a decrease in ID among the animals.

Butyrate acts as an acid-forming agent that has anti-inflammatory and anticarcinogenic properties [2,17]. This additive acts as the main source of energy for colonocytes and enterocytes. Hamer et al [17] reported that butyrate has an antidiarrheal effect and that its therapeutic effect on inflammatory processes in the human colon is already known.

*In vitro* and *in vivo* studies have demonstrated that some plant extracts—among them, tannin extract—exhibited high anti-inflammatory and antimicrobial potential against several gram (+) and gram (−) pathogens, preventing the occurrence of enteritis or intestinal disorders, such as diarrhea [18]. Among these pathogens are *Escherichia coli* and *Salmonella* spp. [11, 19]. Moreover, studies have demonstrated that some herbal extracts increase digestibility and absorption of nutrients, presenting antioxidant activity and improving enzyme activity [13].

Therefore, the piglets that showed the lowest ID may have benefited from the associated action mechanisms of calcium butyrate and the tannin extract, which together may have acted on pathogens present in the intestinal lumen.

### Intestinal morphology

In the morphological analyses, the animals that consumed the diet containing the butyrate- and tannin-based additive treatment showed a lower crypt depth than those in the NC treatment group. During the lactation period, piglets have well-developed villi; however, immediately after weaning, their small intestine undergoes gradual biochemical and histological changes, such as decreased villus height, hyperplasia of crypt cells and increased crypt depth [20]. These changes occur due to the stress caused by separation from the mother, change of environment, mixture with animals of other litters and change of diet, which result in a reduction of digestive and absorptive intestinal functions, directly influencing intestinal health and, consequently, animal growth.

Liu et al [21] used a blend composed of organic acids (citric, fumaric, malic, and sorbic) and essential oils (thymol, vanillin and eugenol) in the diet of broilers (0.30 g/kg) and did not observe differences in villus height, crypt depth, or villus height:crypt depth ratio in the duodenum, but indicated that the intestinal efficiency of digestion and nutrient absorption is related to an adequate morphology of the intestinal epithelium. However, the animals that received the mixture of essential oils and organic acids showed higher villus height and crypt depth in the jejunum than those receiving the NC treatment, corroborating the findings of Zeng et al [13], who tested the effects of essential oils (4.5% cinnamaldehyde and 13.5% thymol) in diets of weanling piglets. In discrepancy, Silva Jr. et al [16] investigated feed additives (benzoic acid and the essential oils of eugenol, thymus, and piperine) in piglet feeding and found no differences compared to the NC group for intestinal histology parameters.

Bilić-Šobot et al [22] provided hydrolyzable tannin at the concentrations of 1%, 2%, and 3% to male pigs in the grower and finisher phases and did not observe differences regarding villus height, crypt depth, villus width or villus height:crypt depth ratio in the jejunum. However, in the duodenum, they observed a significant difference in villus height, where the treatment with the 3% concentration provided higher values than the NC. The authors reinforced the idea that the greater the villus height, the wider the absorption area, and also that in the duodenum there occurs the actuation of pancreatic secretions with food coming from the stomach and that the jejunum is characterized as the site of highest nutrient absorption.

Butyrate production is reduced or even absent in the small intestine, because this organ has reduced microbial population and less substrate available for short-chain fatty acid production [23]. Butyric acid is the main source of energy of colonocytes and plays a crucial role for the intestinal barrier through epithelial maintenance [24], inhibiting apoptosis of the mucosa cells and acting on cell turnover [25,26].

In the digestive tract, butyrate may act directly (upper or lower gastrointestinal tract) or indirectly (small intestine) in tissue development and repair. When added to the diet as a feed additive, butyrate performs the same function in the epithelial cells of the small intestine (enterocytes) [23]. In addition, the lipase, secreted in the duodenum, will enzymatically disrupt the fat coating of a coated product and the calcium-butyrate will be gradually released in comparison with a non-coated product. Once free, calcium-butyrate will dissolve slowly and the butyric acid will be released gradually along the intestinal tract.

In the present study, the treatment containing calcium butyrate + tannin extract resulted in a lower crypt depth in the duodenum of the piglets at 14 d of experimentation, which may indicate less tissue damage, which would be related to a lower inflammatory process [4], and, consequently, no need for excessive cell divisions to replace the enterocytes present in the intestinal villi [21], confirmed by the lower COX-2 immunoexpression for the BT treatment (in the duodenum of piglets at 14 d of experimentation), as discussed below.

### Immunohistochemistry

The lower COX-2 immuno-expression in the BT group at 14 d of the experiment in the duodenum is indicative of reduced tissue injury and lower inflammatory response [1]. Likewise, the lower TNF-α immune-expression for the same group at 35 d of the experiment reveals a reduced presence of macrophages and a possible decrease in the presence of pathogenic microorganisms in the segment. The TNF-α was evaluated as a biomarker for inflammation. The concentration of this biomarker reduced at all times and sites after supplementation of the additive with its anti-inflammatory properties [17]. The absence of significance can be explained by the number of replicates and the variation in the parameter, as the tendency in concentration reduction occurs in all sites and times.

In the jejunum, the reduced COX-2 expression at 14 d for the PC treatment indicated lower inflammatory activity. Rhouma et al [27] explained that colistin sulfate protects the jejunum of piglets against inflammatory processes and increases the use and absorption of available nutrients.

Free radicals are naturally produced in the body or originate due to some biological dysfunction, such as inflammatory processes. The most important compounds are called reactive oxygen species, which are factors for formation of ulcerative and erosive lesions along the gastrointestinal tract [28].

According to study conducted by Barreira et al [4], tannin extract has antioxidant capacity, aiding in the neutralization of free radicals produced during *in vivo* oxidative stress, which may indicate that the antioxidant action of tannin extract helps prevent inflammatory events. It also has the ability to bind to other molecules such as proteins and polysaccharides, forming a protective layer composed of the tannin-protein or tannin-polysaccharides complex, which is associated with a tissue repair process in the case of wounds, burns and inflammation [4].

Butyrate is a product of the animal metabolism itself, produced by the intestinal microbiota, mainly in the colon [25]. It is an important source of energy for colonocytes, aiding in the intestinal barrier function through epithelial maintenance [24], inhibiting apoptosis of mucosal cells, acting in cell turnover [25,26] and contributing to its homeostasis by regulating the expression of genes linked to cellular processes, including cell proliferation and differentiation [2], where the same effects are expected in enterocytes [23].

The inflammatory process occurs as a response to the tissue cell injury, and this process involves a complex cascade of biochemical events such as fluid overflow, enzymatic activation, cell migration, release of mediators, tissue lysis and repair [29].

As a consequence of acute lesion of the intestinal mucosa, three local events take place, resulting in restoration of epithelial continuity and normal permeability, namely, i) contraction of the villi to reduce the exposed surface area, ii) migration of epithelial cells to repair the exposed basal membrane, and iii) closure of the existing spaces between the epithelial cells, which will then be closer together [1].

The activation of the gastrointestinal immune system results in the production of several specialized cells and signaling molecules that play an important role in the regulation of immune and inflammatory responses. Noteworthy examples of such specialized cells and signaling molecules are pro-inflammatory cytokines, such as COX-2 and TNF-α. However, excessive production of those molecules may result in degraded integrity of the intestinal epithelium and consequently affect its functions, e.g., macromolecule permeability and nutrient and ion transport [1,21].

Butyrate can exert immunomodulatory effects, such as ability to suppress the nuclear factor kappa B (NF-κB) [17], a transcription factor responsible for controlling the expression of pro-inflammatory cytokine genes. Therefore, butyrate-induced suppression of NF-κB contributes to reducing COX-2 and TNF-α concentrations [30], which is in line with our findings. In view of the above, a joint effect between tannin extract and calcium butyrate is perceived whereby they act by reducing the immunoexpression of COX-2 and TNF-α.

In conclusion, the use of colistin and the association of calcium butyrate with tannin extracts did not alter the growth performance of weaned piglets, but the association between calcium butyrate and tannin extract resulted in a significant decrease in the incidence of diarrhea and reduced the inflammatory process in the duodenum of piglets in the nursery phase. Thus, butyrate- and tannin-based additive can be a promising alternative for maintaining intestinal health post-weaning and can possibly partly replace antimicrobials used in piglets in the nursery phase.

## Figures and Tables

**Table 1:** Composition and calculated values of the basal diets (as-fed basis)

Items	Pre-starter diet (1 to 14 days)	Starter diet (14 to 35 days)
Ingredient		
Corn (80 g/kg)	435.9	513.0
Soybean meal (460 g/kg)	260.0	250.0
Vitamix^1)^	276.8	202.7
Soy oil	15.0	17.0
Limestone	9.0	9.0
Dicalcium phosphate	-	3.0
L-Lys·HCl (780 g/kg)	2.0	3.8
DL-methionine (990 g/kg)	0.4	0.5
L-threonine (980 g/kg)	0.9	1.0
Calculated value (g/kg)		
Crude protein	197.6	187.6
Ether extract	22.9	23.3
Lactose	60.3	44.1
Lysine	15.1	14.4
Methionine	4.7	4.4
Methionine+cysteine	7.5	7.0
Tryptophan	2.7	2.5
Threonine	9.8	9.2
Metabolizable energy (MJ/kg)	13.99	13.96

1) Vitamix: quantity per kg of product: 155.7 (g/kg) crude protein, 75.9 (g/kg) ether extract, 119.3 (g/kg) mineral material, 5.7 (g/kg) crude fiber, 217.8 (g/kg) lactose, 54.3 (g/kg) milk protein, 16.4 (g/kg) calcium, 14.9 (g/kg) total phosphorus, 13.4 (g/kg) available phosphorus, 12.2 (g/kg) sodium, 13.9 (g/kg) potassium, 20.0 (g/kg) chlorine, 16.9 (g/kg) lysine, 7.0 (g/kg) methionine, 9.6 (g/kg) methionine+cystine, 2.9 (g/kg) tryptophan, 10.3 (g/kg) threonine, 8.0 (g/kg) arginine, 13.0 (g/kg) leucine, 7.2 (g/kg) isoleucine, 3.6 (g/kg) histidine, 7.5 (g/kg) valine, 6.3 (g/kg) phenylalanine, 14.12 MJ/kg of metabolizable energy, 50,000 IU of vitamin A, 10,000 IU of vitamin D_3_, 160 mg vitamin E, 12 mg vitamin K_3_, 12 mg vitamin B_1_, 20 mg vitamin B_2_, 12 mg vitamin B_6_, 0.10 mg vitamin B_12_, 2.40 mg folic acid, 140 mg nicotinic acid, 88 mg pantothenic acid, 0.40 mg biotin, 1,248 mg choline, 800 mg iron sulfate, 800 mg copper sulfate, 220 mg manganese sulfate, 3.20 mg cobalt sulfate, 5,000 mg zinc oxide, 7.20 mg iodine, 1.20 mg sodium selenite, 1.20 mg chromium sulfate.

**Table 2:** Mean values for the experimental periods of 1 to 14 and 1 to 35 days

Variable	Treatment^1)^				

	NC	PC	BT	SEM	p-value^*^
1 to 14 days					
BW1 (kg)	7.00	7.26	7.20	-	-
BW14 (kg)	11.87	12.09	11.92	0.30	0.787
ADG (kg)	0.35	0.34	0.34	0.01	0.871
ADFI (kg)	0.47	0.48	0.45	0.01	0.304
G:F	0.76	0.71	0.75	0.02	0.434
ID (%)^2)^	11.63^a^	14.61^a^	8.35^b^	-	-
1 to 35 days					
BW35 (kg)	25.05	26.81	25.48	0.74	0.553
ADG (kg)	0.52	0.56	0.52	0.02	0.617
ADFI (kg)	0.84	0.88	0.83	0.02	0.561
G:F	0.62	0.64	0.63	0.01	0.510
ID (%)^2)^	6.75^a^	8.26^a^	4.88^b^	-	-

SEM, standard error of the mean; BW1, body weight; BW14, body weight at 14 days; ADG, average daily gain; ADFI, average daily feed intake; G:F, gain to feed ratio; ID, incidence of diarrhea; BW35, body weight at 35 days.

1) NC, negative control: basal diet; PC, positive control: basal diet + colistin sulfate; BT, basal diet + butyrate- and tannin-based additive.

2) ID (%) = total number of feces counted daily in the pen.

* Diet effect.

a,b Different letters in the same row are statistically different by the Chi-square test (p<0.05).

**Table 3:** Mean values of intestinal morphometry in the duodenum and jejunum of piglets at 14 and 35 days of experiment

Variable	Treatment^1)^									

	Day 14					Day 35				
	
	NC	PC	BT	SEM	p-value^*^	NC	PC	BT	SEM	p-value^*^
	------------------------------ Duodenum ---------------------------------					----------------------------------- Duodenum ---------------------------------------				
VH (μm)	447	386	341	20.49	0.098	508	413	425	26.61	0.335
CD (μm)	422^a^	393^ab^	303^b^	20.51	0.025	460	400	417	22.66	0.595
VH:CD	1.06	1.00	1.14	0.06	0.692	1.12	1.06	1.06	0.07	0.926
VW (μm)	154	152	131	6.13	0.24	173	153	216	18.99	0.390
VD (μm^2^)	38.35	33.35	32.00	2.72	0.667	31.70	22.75	27.70	2.75	0.451
	------------------------------------ Jejunum ------------------------------------					----------------------------------------- Jejunum ------------------------------------				
VH (μm)	343	379	341	20.95	0.749	433	376	406	26.19	0.710
CD (μm)	358	333	303	12.53	0.218	363	314	314	12.69	0.215
VH:CD	1.01	1.14	1.14	0.09	0.817	1.22	1.2	1.32	0.09	0.876
VW (μm)	160	135	131	7.5	0.23	153	136	158	7.25	0.464
VD (μm^2^)	43.25	32.70	35.00	5.23	0.73	18.75	22.00	26.75	2.11	0.328

SEM, standard error of the mean; VH, villus height; CD, crypt depth; VH:CD, villus height:crypt depth; VW, villus width; VD, villus density.

1) NC, negative control: basal diet; PC, positive control: basal diet + colistin sulfate; BT, basal diet + butyrate- and tannin-based additive.

* Diet effect.

a,b Different letters in the same row are statistically different (p<0.05) according to Tukey’s test.

**Table 4:** Immunohistochemistry in the duodenum and jejunum of piglets at 14 days and 35 days of experiment

Variable	Treatment^1)^				

	NC	PC	BT	SEM	p-value^*^
COX-2 (%)					
14 days					
Duodenum	6.89^a^	5.59^ab^	2.75^b^	0.65	0.011
Jejunum	3.76	1.98	3.54	0.52	0.346
35 days					
Duodenum	3.30	5.05	5.28	0.42	0.097
Jejunum	1.83^a^	1.49^b^	1.86^a^	0.06	0.020
TNF-α (average counting)					
14 days					
Duodenum	65.05	53.33	27.05	8.27	0.161
Jejunum	67.47	53.50	48.34	6.51	0.508
35 days					
Duodenum	76.20^a^	63.82^ab^	32.49^b^	7.52	0.029
Jejunum	64.96	39.74	34.86	7.95	0.273

SEM, standard error of the mean; COX-2, cyclooxygenase-2; TNF-α, tumor necrosis factor α.

1) NC = negative control: basal diet, PC = positive control: basal diet + colistin sulfate, BT = basal diet + butyrate- and tannin-based additive.

* Diet effect.

a,b Different letters in the same row are statistically different (p<0.05) according to Tukey’s test.

## References

[1] Liu Y (2015). Fatty acids, inflammation and intestinal health in pigs. J Anim Sci Biotechnol.

[2] Celasco G, Moro L, Aiello C (2014). Calcium butyrate: Anti-inflammatory effect on experimental colitis in rats and antitumor properties. Biomed Rep.

[3] Nazari M, Karkoodi K, Alizadeh A (2012). Performance and physiological responses of milk-fed calves to coated calcium butyrate supplementation. S Afr J Anim Sci.

[4] Barreira JCM, Ferreira ICFR, Oliveira MBPP, Pereira JA (2008). Antioxidant activities of the extracts from chestnut flower, leaf, skins and fruit. Food Chem.

[5] Rostagno HS, Albino LFT, Donzele JL (2011). Brazilian tables for poultry and swine: composition of feedstuffs and nutritional requirements.

[6] Salles MSV, Zanetti MA, Roma LCJ (2014). Performance and immune response of suckling calves fed organic selenium. Anim Feed Sci Technol.

[7] Touchette KJ, Carroll JA, Allee GL (2002). Effect of spray-dried plasma and lipopolysaccharide exposure on weaned pigs: effects on the immune axis of weaned pigs. J Anim Sci.

[8] Araujo JC, Téran FC, Oliveira RA (2003). Comparison of hexamethyldisilazane and critical point drying treatments for SEM analysis of anaerobic biofilms and granular sludge. J Electron Microsc.

[9] Engracia Filho JR, Araújo CD, Pinto GN, Mendes YH, Bechara GH (2017). Cellular response in the tick feeding site in crossbred cattle artificially infested by Rhipicephalus microplus. Exp Appl Acarol.

[10] Costa Filho OAAD, Ribas Filho JM, Ariede BL, Cavalcanti T, Scapini JGS, Pasetto CV (2017). Comparative efficacy of immunohistochemical markers in surgical healing. Rev Col Bras Cir.

[11] Biagi G, Cipollini I, Paulicks BR, Roth FX (2010). Effect of tannins on growth performance and intestinal ecosystem in weaned piglets. Arch Anim Nutr.

[12] Boas ADCV, Budiño FEL, Neto MAT (2016). Organic acids in diets of weaned piglets: Performance, digestibility and economical viability. Arq Bras Med Vet Zootec.

[13] Zeng Z, Xu X, Zhang Q (2015). Effects of essential oil supplementation of a low-energy diet on performance, intestinal morphology and microflora, immune properties and antioxidant activities in weaned pigs. Anim Sci.

[14] Costa LB, Tse MLP, Miyada VS (2007). Herbal extracts as alternatives to antimicrobial growth promoters for weanling pigs. R Bras Zootec.

[15] Cairo PLG, Gois FD, Sbardella M (2018). Effects of dietary supplementation of red pepper (Schinus terebinthifolius Raddi) essential oil on performance, small intestinal morphology and microbial counts of weanling pigs. J Sci Food Agric.

[16] Silva CD, Martins CCS, Dias FTF (2017). Alternative feed additive, associated or not with antibiotic, in weaned piglets feeding: Diarrhea incidence, intestinal mucosa morphology, and digestive organs weight. J Anim Sci.

[17] Hamer HM, Jonkers D, Venema K, Vanhoutvin S, Troost FJ, Brummer RJ (2008). Review article: The role of butyrate on colonic function. Aliment Pharmacol Ther.

[18] Hanczakowska E, Swiatkiewicz M (2012). Effect of herbal extracts on piglet performance and small intestinal epithelial villi. Czech J Anim Sci.

[19] Thacker PA (2013). Alternatives to antibiotics as growth promoters for use in swine production: a review. J Anim Sci Biotechnol.

[20] Pluske JR (2016). Invited review: Aspects of gastrointestinal tract growth and maturation in the pre- and postweaning period of pigs. J Anim Sci.

[21] Liu Y, Yang X, Xin H (2017). Effects of a protected inclusion of organic acids and essential oils as antibiotic growth promoter alternative on growth performance, intestinal morphology and gut microflora in broilers. Anim Sci J.

[22] Bilić-Šobot D, Kubale V, Škrlep M (2016). Effect of hydrolysable tannins on intestinal morphology, proliferation and apoptosis in entire male pigs. Arch Anim Nutr.

[23] Claus R, Günthner D, Letzguß H (2007). Effects of feeding fat-coated butyrate on mucosal morphology and function in the small intestine of the pig. J Anim Physiol Anim Nutr.

[24] Plöger S, Stumpff F, Penner GB (2012). Microbial butyrate and its role for barrier function in the gastrointestinal tract. Ann NY Acad Sci.

[25] Bartholome AL, Albin DM, Baker DH, Holst JJ, Tappenden KA (2004). Supplementation of total parenteral nutrition with butyrate acutely increases structural aspects of intestinal adaptation after an 80% jejunoileal resection in neonatal piglets. J Parenter Enteral Nutr.

[26] Woodward AD, Regmi PR, Gänzle MG, Van Kempen TATG, Zijlstra RT (2012). Slowly digestible starch influences mRNA abundance of glucose and short-chain fatty acid transporters in the porcine distal intestinal tract. J Anim Sci.

[27] Rhouma M, Fairbrother JM, Beaudry F, Letellier A (2017). Post weaning diarrhea in pigs: risk factors and non-colistin-based control strategies. Acta Vet Scand.

[28] Muss C, Mosgoeller W, Endler T (2013). Papaya preparation (Caricol^®^) in digestive disorders. Neuro Endocrinol Lett.

[29] Carvalho WA, Lemonica L (1998). Molecular and cellular mechanisms of inflammatory pain. Peripheral modulation and therapeutic advances. Rev Bras Anestesiol.

[30] Song M, Xia B, Li J (2006). Effects of topical treatment of sodium butyrate and 5-aminosalicylic acid on expression of trefoil factor 3, interleukin 1β, and nuclear factor κB in trinitrobenzene sulphonic acid induced colitis in rats. Postgrad Med J.

